# Association between CASP8 –652 6N Del Polymorphism (rs3834129) and Colorectal Cancer Risk: Results from a Multi-Centric Study

**DOI:** 10.1371/journal.pone.0085538

**Published:** 2014-01-21

**Authors:** Barbara Pardini, Paolo Verderio, Sara Pizzamiglio, Carmela Nici, Maria Valeria Maiorana, Alessio Naccarati, Ludmila Vodickova, Veronika Vymetalkova, Silvia Veneroni, Maria Grazia Daidone, Fernando Ravagnani, Tiziana Bianchi, Luis Bujanda, Angel Carracedo, Antoni Castells, Clara Ruiz-Ponte, Hans Morreau, Kimberley Howarth, Angela Jones, Sergi Castellví-Bel, Li Li, Ian Tomlinson, Tom Van Wezel, Pavel Vodicka, Paolo Radice, Paolo Peterlongo, the EPICOLON Consortium

**Affiliations:** 1 Genomic Variation in Human Populations and Complex Diseases Unit, Human Genetics Foundation, Turin, Italy; 2 Unit of Medical Statistics, Biometry and Bioinformatics, Fondazione Istituto di Ricovero e Cura a Carattere Scientifico Istituto Nazionale dei Tumori, Milan, Italy; 3 Fondazione Istituto Italian Foundation for Cancer Research di Oncologia Molecolare, Milan, Italy; 4 Molecular and Genetic Epidemiology Unit, Human Genetics Foundation, Turin, Italy; 5 Department of Molecular Biology of Cancer, Institute of Experimental Medicine, Academy of Sciences of the Czech Republic, Prague, Czech Republic; 6 First Medical Faculty, Charles University, Prague, Czech Republic; 7 Department of Experimental Oncology and Molecular Medicine, Fondazione Istituto di Ricovero e Cura a Carattere Scientifico Istituto Nazionale dei Tumori, Milan, Italy; 8 Immunohematology and Transfusion Medicine Service, Fondazione Istituto di Ricovero e Cura a Carattere Scientifico Istituto Nazionale dei Tumori, Milan, Italy; 9 Associazione Italiana Volontari Sangue Comunale Milano, Milan, Italy; 10 Gastroenterology Department, Hospital Donostia, Networked Biomedical Research Centre for Hepatic and Digestive Diseases, Basque Country University, San Sebastián, Spain; 11 Galician Public Foundation of Genomic Medicine, Centro de Investigación Biomédica en Red de Enfermedades Raras, Genomics Medicine Group, Hospital Clínico, Santiago de Compostela, University of Santiago de Compostela, Galicia, Spain; 12 Department of Gastroenterology, Hospital Clínic, Centro de Investigación Biomédica en Red de Enfermedades Hepáticas y Digestivas, Institut d'Investigacions Biomèdiques August Pi i Sunyer, University of Barcelona, Barcelona, Catalonia, Spain; 13 Department of Pathology, Leiden University Medical Center, Leiden, The Netherlands; 14 Molecular and Population Genetics Laboratory and National Institute for Health Research Comprehensive Biomedical Research Centre, Wellcome Trust Centre for Human Genetics, University of Oxford, Oxford, United Kingdom; 15 Department of Family Medicine and Community Health and Case Comprehensive Cancer Center, Case Western Reserve University, Cleveland, Ohio, United States of America; 16 Unit of Molecular Bases of Genetic Risk and Genetic Testing, Department of Preventive and Predictive Medicine, Fondazione Istituto di Ricovero e Cura a Carattere Scientifico Istituto Nazionale dei Tumori, Milan, Italy; Duke Cancer Institute, United States of America

## Abstract

The common −652 6N del variant in the *CASP8* promoter (rs3834129) has been described as a putative low-penetrance risk factor for different cancer types. In particular, some studies suggested that the deleted allele (del) was inversely associated with CRC risk while other analyses failed to confirm this. Hence, to better understand the role of this variant in the risk of developing CRC, we performed a multi-centric case-control study. In the study, the variant −652 6N del was genotyped in a total of 6,733 CRC cases and 7,576 controls recruited by six different centers located in Spain, Italy, USA, England, Czech Republic and the Netherlands collaborating to the international consortium COGENT (COlorectal cancer GENeTics). Our analysis indicated that rs3834129 was not associated with CRC risk in the full data set. However, the del allele was under-represented in one set of cases with a family history of CRC (per allele model OR = 0.79, 95% CI = 0.69–0.90) suggesting this allele might be a protective factor versus familial CRC. Since this multi-centric case-control study was performed on a very large sample size, it provided robust clarification of the effect of rs3834129 on the risk of developing CRC in Caucasians.

## Introduction

Carcinogenesis is characterized by the alteration of the normal processes designated to maintain the genome stability. Apoptosis is the most prominent mechanism of the programmed cell-death, responsible for the safe removal of damaged cells before genome abnormalities can be replicated and further spread. Caspase enzymes are essential in the regulation and execution of most of the apoptotic cell-death pathways. In particular, caspase-8 (*CASP8*) is a crucial player in controlling the apoptosis of T lymphocytes through activation-induced cell-death [1] and, simultaneously, a physiological homeostasis of T lymphocytes is a fundamental aspect in the immune-surveillance of cancer cells.

A polymorphism consisting of the deletion of six nucleotides in the promoter region of *CASP8*, and referred as –652 6N del (rs3834129), has been described to be very common in several populations [2]. This six-nucleotide deletion was shown to destroy a stimulatory protein 1 binding element in the promoter regulatory region that causes a decreased *CASP8* transcription and eventually a reduced apoptosis of antitumor T lymphocytes [2]. Thus, rs3834129 was postulated to affect the antitumor immune response during cancer initiation or progression, and consequently considered as a genetic factor potentially associated with cancer risk. In this light, the polymorphism was tested in a case-control study and the del allele was shown to be associated with a protective effect in several types of cancer, including CRC, in the Chinese population [2]. Two subsequent studies further investigated the effect of rs3834129 in CRC testing cases and controls of mixed and Caucasian ethnicity but failed to confirm the association [3], [4]. A meta-analysis of these three studies indicated that, under a dominant model, the del allele was associated with a significantly reduced risk for CRC with odds ratio (OR) = 0.89, 95% confidence interval (CI) = 0.83–0.96 [5]. However, a later study not included in the above meta-analysis, again failed to reveal an association between rs3834129 and CRC risk in Chinese [6].

Hence, in the present study we sought for more robust proof, whether rs3834129 may be a CRC risk factor in a case-control study, based on six cohorts recruited in centers located in Spain, Italy, USA, England, Czech Republic and the Netherlands and collaborating within the COGENT (COlorectal cancer GENeTics) consortium [7].

## Materials and Methods

### Case-control cohorts

The COGENT (COlorectal cancer GENeTics) consortium was established in 2007 with the main goal to study genetic susceptibility to CRC in a collaborative way. The consortium consisted in over 20 research groups in Europe, Australia, the Americas, China and Japan actively working on CRC genetics and with expertise encompassing genetic epidemiology, statistical genetics, gene mapping, biology, molecular genetics, pathology and diagnosis and the clinical management of CRC [8]. Maintaining its main objectives, the consortium has now evolved into a more structured initiative named “Cooperation Studies on Inherited Susceptibility to Colorectal Cancer” (EuCOLONGENE - http://www.eucolongene.eu). In the present study, rs3834129 was tested as genetic risk factor for CRC in six cohorts comprising 6,733 cases and 7,576 controls.

1. Spanish cohort. Cases and controls were recruited through the EPICOLON Consortium that is based on a prospective, multicenter and population-based epidemiology survey of the incidence and features of CRC in the Spanish population [9]. Briefly, cases were selected as patients with *de novo* histologically confirmed diagnosis of colorectal adenocarcinoma. Exclusion criteria were hereditary CRC forms, such as hereditary non-polyposis colorectal cancer (HNPCC) and familial adenomatous polyposis (FAP) and a personal history of inflammatory bowel disease. Controls were from the Spanish National DNA bank and were confirmed not to have cancer or history of neoplasm and no family history of CRC. All cases and controls were of Caucasian ethnicity. 2. Italian cohort. Cases and controls were recruited as described by [10]. Briefly, the cases were consecutive individuals affected with CRC who underwent surgery at the Fondazione IRCCS Istituto Nazionale Tumori in Milan (INT). The controls were blood donors recruited through the Immunohematology and Transfusion Medicine Service of INT the Associazione Volontari Italiani Sangue Comunale in Milan. All cases and controls were of Caucasian ethnicity. 3. American cohort (Kentucky, USA). Cases and controls were recruited as recently described [11]. Briefly, incident colon cancer cases were identified through the Kentucky Cancer Registry. Population controls were recruited via random digit dialing according to the following criteria: being at least 30 years of age or older and free of personal history of cancer other than skin cancer. For both cases and controls, exclusion criteria were inflammatory bowel diseases, FAP and HNPCC. Majority of the participants were Caucasians (93.7%). 4. English cohort. Cases (CRC or significant adenomas) and controls were recruited through Colorectal Tumour Gene Identification (CoRGI) consortium as previously described [12]. Briefly, cases had at least one first-degree relative affected by CRC. The controls were spouses or partners unaffected by cancer and without a personal and family history of colorectal neoplasia. A single proband from each family was included in this study. Hereditary CRC forms such as, HNPCC/Lynch syndrome or bi-allelic MutYH mutation carriers were excluded. All cases and controls were of Caucasian ethnicity. 5. Czech Republic cohort. Cases and controls were recruited as previously described [13]. Briefly, all cases had histologically confirmed CRC and were consecutively ascertained through oncological departments. Controls were either hospital-based volunteers with negative colonoscopy results or blood donors collected from a blood donor center in Prague. All cases and controls were of Caucasian ethnicity. 6. Dutch Cohort. Cases and controls were recruited as previously described [14]. Briefly, most of the cases were recruited through the clinical genetics department. All cases were diagnosed with CRC and had early onset and/or positive family history for CRC. Known dominant polyposis syndromes, HNPCC/Lynch syndrome or bi-allelic MutYH mutation carriers were excluded. A single proband from each family was included in this study. Controls were healthy blood donors from the southwest region of the Netherlands. All cases and controls were of Caucasian ethnicity.

For each cohort, number of cases and controls included in the study and their sex and age data are shown in Table 1. All individuals participating in this study signed an informed consent to the use of their biological samples for research purposes. This study was approved by the following Institutions: Clinical Research Ethics and Research Committees of the Hospital Clínic in Barcelon (Spanish cohort); Ethics Committee of Fondazione IRCCS Istituto Nazionale dei Tumori, Milan, Italy (Italian cohort); Institutional Review Boards of the University of Kentucky, Lexington, Case Western Reserve University/University Hospitals of Cleveland and University of Southern California, USA (American cohort); Southampton and South-West Hampshire Research Ethics Committee (English cohort); Ethics Committee of the Institute of Experimental Medicine, and Ethics Committee of the Institute for Clinical and Experimental Medicine and Thomayer Hospital, Prague, Czech Republic (Czech Republic cohort); the Medical Ethical Committee of the Leiden University Medical Center, The Netherlands (protocol P01.019) (Dutch cohort).

**Table 1:** Characteristics of the cohorts included in the study.

Cohort	Group	N		Age		Female (%)	Male (%)
			min	median	max		
Spanish	Case	1978	26	72	101	778 (39.3)	1200 (60.7)
	Control	1647	19	66	95	748 (45.4)	899 (54.6)
Italian	Case	617	24	64	91	243 (39.4)	374 (60.6)
	Control	2551	18	44	71	1619 (63.5)	932 (36.5)
USA	Case	1010	23	65	90	501 (49.6)	509 (50.4)
	Control	1580	31	61	91	1017 (64.4)	563 (35.6)
English	Case	1576	18	65	89	712 (45.2)	864 (54.8)
	Control	767	18	56	86	462 (60.2)	305 (39.8)
Czech Rep.	Case	967	26	62	89	393 (40.6)	574 (59.4)
	Control	672	24	57	91	314 (46.7)	358 (53.3)
Dutch	Case	585*	19	52	90	293 (50.1)	261 (44.6)
	Control	359		NA		NA	NA

* Data on sex were missing for 31 cases.

### Genotyping

The genomic DNA was isolated from peripheral blood lymphocytes using standard procedures. The DNA samples from cases and controls were randomly aliquot in 96-well plates. Genotyping of the rs3834129 was carried out by using the Taqman assay (Life Technologies/Applied Biosystems, US) in the Spanish, Italian, USA and Dutch cohorts and using the KASPar assay (K-bioscience, UK) in the English and Czech Republic cohorts. Duplicate samples (5%), no template controls in each plate, and Hardy–Weinberg equilibrium test were used as quality control tests.

### Statistical analysis

Within each cohort, a logistics regression analysis [15] was carried out in order to compare the genotypes frequency of *CASP8* rs3834129 in cases and controls. We estimated the odds ratio (OR) and their relative 95% confidence interval (CI) by considering in each logistic model age and sex as adjustment covariates. Four different models (“three genotypes”, dominant, recessive and per allele) were performed for each cohort separated and considering all the individuals together. In this case, the variable with the indication of the cohort and the interaction term between this variable and the genotype were included in the logistic model in addition to the variables genotype, age and sex. The deviations of the genotype frequencies in the controls from those expected under Hardy-Weinberg equilibrium (HWE) were assessed within each cohort as well as by considering all individuals using Pearson's chi-squared test. All statistical analyses were performed with the SAS software (Version 9.2; SAS Institute Inc. Cary, NC).

## Results and Discussion

In this study, we analyzed the rs3834129 genotype distribution in a total of 6,733 cases and 7,576 controls from six cohorts, all of Caucasian ethnicity. The genotype distributions in controls were consistent with HWE in all the cohorts and across them the frequencies of the del allele were similar in controls (range: 0.45–0.52). We performed an overall analysis to compare the genotypes frequency of *CASP8* rs3834129 in cases with CRC and in healthy controls. As in the Dutch controls data on sex and/or age were missing (Table 1), this cohort was excluded from the overall analysis and a total of 6,148 cases and 7,217 controls were considered. All cohorts were based on incident/consecutive cases except for the English cohort which was based on familial cases. Therefore, we also performed an additional overall analysis excluding the English cohort and testing a total of 4,572 cases and 6,450 controls. In both these analyses the interaction term between cohort and genotype was not statistically significant and also the ORs derived from the different implemented genetic models were not statistically significant (data not shown).

Furthermore, we assessed the effect of rs3834129 on CRC risk within each cohort. The OR estimates were adjusted for sex and age with the only exception of Dutch cohort, results are reported in Table 2. The ORs of the different implemented models (three genotypes, dominant, recessive and per-allele model) largely confirmed the results of the overall analysis being non statistically significant in each cohort with the only exception of the English one. The ORs in familial case-control samples from England were statistically significant (per allele model OR = 0.79, 95% CI = 0.69–0.90) indicating the del allele might be a protective factor versus familial cancer. This result may be explained considering that the familial cases, with respect to incident/consecutive cases, can be a better resource for association testing since they are expected to be enriched by genetic risk factors, or conversely, deprived by factors with a protective effect.

**Table 2:** Genotype/allele frequencies of rs3834129 SNP in COGENT consortium cohorts and risk of CRC (logistic regression analysis).

Cohort	Genetic model	Case (%)	Control (%)	OR	95% CI	p-value
Spanish	nor/nor	500 (25.3)	425 (25.8)	1,00		
	nor/del	996 (50.3)	802 (48.7)	1,06	0.90–1.26	0,452
	del/del	482 (24.4)	420 (25.5)	0,96	0.79–1.16	0,651
	Dominant			1,03	0.88–1.20	0,730
	Recessive			0,92	0.78–1.07	0,283
	Allelic			0,98	0.89–1.08	0,658
Italian	nor/nor	195 (31.6)	783 (30.7)	1,00		
	nor/del	285 (46.2)	1230 (48.2)	0,93	0.72–1.21	0,609
	del/del	137 (22.2)	538 (21.1)	0,85	0.62–1.16	0,313
	Dominant			0,91	0.71–1.15	0,432
	Recessive			0,89	0.68–1.16	0,385
	Allelic			0,92	0.79–1.08	0,306
USA	nor/nor	237 (23.5)	383 (24.2)	1,00		
	nor/del	514 (50.9)	794 (50.2)	1,07	0.88–1.31	0,501
	del/del	259 (25.6)	403 (25.5)	1,04	0.83–1.32	0,678
	Dominant			1,06	0.88–1.28	0,521
	Recessive			1,00	0.83–1.20	0,984
	Allelic			1,02	0.91–1.15	0,689
English	nor/nor	410 (26.0)	165 (21.5)	1,00		
	nor/del	825 (52.4)	393 (51.2)	0,79	0.62–1.00	0,051
	del/del	341 (21.6)	209 (27.3)	0,61	0.47–0.81	0,001
	Dominant			0,73	0.58–0.91	0,006
	Recessive			0,72	0.58–0.90	0,003
	Allelic			0,79	0.69–0.90	0,0006
Czech Rep.	nor/nor	239 (24.7)	169 (25.1)	1,00		
	nor/del	479 (49.5)	326 (48.5)	1,04	0.82–1.34	0,724
	del/del	249 (25.7)	177 (26.3)	0,98	0.74–1.31	0,922
	Dominant			1,02	0.81–1.29	0,838
	Recessive			0,96	0.76–1.20	0,708
	Allelic			0,99	0.86–1.14	0,915
Dutch	nor/nor	169 (28.9)	106 (29.5)	1,00		
	nor/del	282 (48.2)	177 (49.3)	1,00	0.74–1.36	0,996
	del/del	134 (22.9)	76 (21.2)	1,11	0.76–1.60	0,596
	Dominant			1,03	0.77–1.38	0,834
	Recessive			1,11	0.80–1.52	0,534
	Allelic			1,05	0.87–1.26	0,616

To our knowledge, this is the largest analysis testing the association between rs3834129 and CRC risk in Caucasians and our data provide the stronger and unambiguous evidence so far that the rs3834129 is not a CRC risk factor in this ethnic group. While our analysis was ongoing, other studies appeared, showing inconsistent results. On one side, lack of association was found in case-control analyses based on Greek and Chinese populations [16], [17]; on the other side, a meta-analysis, based on three studies of mix ethnicity, and a separate additional study on Chinese showed a moderately protective effect of the del allele [18], [19]. Our findings in Caucasians, with respect to the putative protective effect detected in Chinese—that has to be confirmed by further larger studies—might be explained by additional risk-associated variants in linkage with rs3834129 and with different frequency in different ethnic genetic backgrounds. Specifically, the del allele of rs3834129 has a 0.48 control frequency in our study while we derived a frequency of 0.20–0.25 in Chinese controls [2], [6], [16], [18].

In conclusion, while further studies are needed to confirm the protective effect of the del allele we observed in familial CRC cases with family history, our study provides robust evidence indicating the rs3834129 is not a risk factor for CRC in Caucasians.
