# Correction: Association between CASP8 –652 6N Del Polymorphism (rs3834129) and Colorectal Cancer Risk: Results from a Multi-Centric Study

**DOI:** 10.1371/journal.pone.0091310

**Published:** 2014-03-07

**Authors:** The PLOS ONE staff

There are multiple errors in the order and affiliations of multiple authors. Please see the correct author order and list of affiliations below.

Barbara Pardini^1^, Paolo Verderio^2^, Sara Pizzamiglio^2^, Carmela Nici^3^, Maria Valeria Maiorana^3^, Alessio Naccarati^1,4^, Ludmila Vodickova^5,6^, Veronika Vymetalkova^5,6^, Silvia Veneroni^7^, Maria Grazia Daidone^7^, Fernando Ravagnani^8^, Tiziana Bianchi^9^, EPICOLON Consortium^¶^, Luis Bujanda^10^, Angel Carracedo^11^, Antoni Castells^12^, Clara Ruiz-Ponte^11^, Hans Morreau^13^, Kimberley Howarth^14^, Angela Jones^14^, Sergi Castellví-Bel^12^, Li Li^15^, Ian Tomlinson^14^, Tom Van Wezel^13^, Pavel Vodicka^4,5^, Paolo Radice^16^, Paolo Peterlongo^3^

1. Genomic Variation in Human Populations and Complex Diseases Unit, Human Genetics Foundation, Turin, Italy.
2. Unit of Medical Statistics, Biometry and Bioinformatics, Fondazione IRCCS Istituto Nazionale dei Tumori, Milan, Italy.
3. IFOM, Fondazione Istituto FIRC di Oncologia Molecolare, Milan, Italy.
4. Molecular and Genetic Epidemiology Unit, Human Genetics Foundation, Turin, Italy.
5. Department of Molecular Biology of Cancer, Institute of Experimental Medicine, Academy of Sciences of the Czech Republic, Prague, Czech Republic.
6. First Medical Faculty, Charles University, Prague, Czech Republic.
7. Department of Experimental Oncology and Molecular Medicine, Fondazione IRCCS Istituto Nazionale dei Tumori, Milan, Italy.
8. Immunohematology and Transfusion Medicine Service, Fondazione IRCCS Istituto Nazionale dei Tumori, Milan, Italy.
9. Associazione Italiana Volontari Sangue (AVIS) Comunale Milano, Milan, Italy.
10. Gastroenterology Department, Hospital Donostia, Networked Biomedical Research Centre for Hepatic and Digestive Diseases (CIBEREHD), Basque Country University, San Sebastián, Spain.
11. Galician Public Foundation of Genomic Medicine (FPGMX), Centro de Investigación Biomédica en Red de Enfermedades Raras (CIBERER), Genomics Medicine Group, Hospital Clínico, Santiago de Compostela, University of Santiago de Compostela, Galicia, Spain.
12. Department of Gastroenterology, Hospital Clínic, Centro de Investigación Biomédica en Red de Enfermedades Hepáticas y Digestivas (CIBEREHD), Institut d’Investigacions Biomèdiques August Pi i Sunyer (IDIBAPS), University of Barcelona, Barcelona, Catalonia, Spain.
13. Department of Pathology, Leiden University Medical Center, Leiden, The Netherlands.
14. Molecular and Population Genetics Laboratory and NIHR Comprehensive Biomedical Research Centre, Wellcome Trust Centre for Human Genetics, University of Oxford, Oxford, United Kingdom.
15. Department of Family Medicine and Community Health and Case Comprehensive Cancer Center, Case Western Reserve University, Cleveland, Ohio, United States of America.
16. Unit of Molecular Bases of Genetic Risk and Genetic Testing, Department of Preventive and Predictive Medicine, Fondazione IRCCS Istituto Nazionale dei Tumori, Milan, Italy.

¶ Please see the Acknowledgments for a full list of authors comprising the EPICOLON Consortium and their respective affiliations.

In addition, there are multiple errors in Table 2. Please see a corrected version of Table 2 here:

**Table 2:** Genotype/allele frequencies of rs3834129 SNP in COGENT consortium cohorts and risk of CRC (logistic regression analysis).

Cohort	Genetic model	Case (%)	Control (%)	OR	95% CI	p-value
Spanish	nor/nor	500 (25.3)	425 (25.8)	1.00		
	nor/del	996 (50.3)	802 (48.7)	1.06	0.90−1.26	0.452
	del/del	482 (24.4)	420 (25.5)	0.96	0.79−1.16	0.651
	Dominant			1.03	0.88−1.20	0.730
	Recessive			0.92	0.78−1.07	0.283
	Allelic			0.98	0.89−1.08	0.658
						
Italian	nor/nor	195 (31.6)	783 (30.7)	1.00		
	nor/del	285 (46.2)	1230 (48.2)	0.93	0.72−1.21	0.609
	del/del	137 (22.2)	538 (21.1)	0.85	0.62−1.16	0.313
	Dominant			0.91	0.71−1.15	0.432
	Recessive			0.89	0.68−1.16	0.385
	Allelic			0.92	0.79−1.08	0.306
						
USA	nor/nor	237 (23.5)	383 (24.2)	1.00		
	nor/del	514 (50.9)	794 (50.2)	1.07	0.88−1.31	0.501
	del/del	259 (25.6)	403 (25.5)	1.04	0.83−1.32	0.678
	Dominant			1.06	0.88−1.28	0.521
	Recessive			1.00	0.83−1.20	0.984
	Allelic			1.02	0.91−1.15	0.689
						
English	nor/nor	410 (26.0)	165 (21.5)	1.00		
	nor/del	825 (52.4)	393 (51.2)	0.79	0.62−1.00	0.051
	del/del	341 (21.6)	209 (27.3)	0.61	0.47−0.81	0.001
	Dominant			0.73	0.58−0.91	0.006
	Recessive			0.72	0.58−0.90	0.003
	Allelic			0.79	0.69−0.90	0.0006
						
Czech Rep.	nor/nor	239 (24.7)	169 (25.1)	1.00		
	nor/del	479 (49.5)	326 (48.5)	1.04	0.82−1.34	0.724
	del/del	249 (25.7)	177 (26.3)	0.98	0.74−1.31	0.922
	Dominant			1.02	0.81−1.29	0.838
	Recessive			0.96	0.76−1.20	0.708
	Allelic			0.99	0.86−1.14	0.915
						
Dutch	nor/nor	169 (28.9)	106 (29.5)	1.00		
	nor/del	282 (48.2)	177 (49.3)	1.00	0.74−1.36	0.996
	del/del	134 (22.9)	76 (21.2)	1.11	0.76−1.60	0.596
	Dominant			1.03	0.77−1.38	0.834
	Recessive			1.11	0.80−1.52	0.534
	Allelic			1.05	0.87−1.26	0.616
